# A scientometric review of global research on sustainability and project management dataset

**DOI:** 10.1016/j.dib.2019.104312

**Published:** 2019-07-31

**Authors:** Roberto Farias de Toledo, H.L. Miranda Junior, J.R. Farias Filho, Helder Gomes Costa

**Affiliations:** Department of Engineering, Federal Fluminense University, Niterói, Rio de Janeiro, Brazil

**Keywords:** Sustainable project, Project management, Green project, Sustainability, Triple bottom line

## Abstract

There are few works that have attempted to map the global research on sustainability and project management. This research utilizes scientometric review of global sustainability and project management research in 2006–2018, through co-word analysis, co-author analysis, journal analysis, institution analysis, and country analysis. A total of 400 bibliographic records from the Web of Science and Scopus core collection databases were selected and analyzed. The findings reveal an evolution of the research field based on the concepts in the Brundtland Commission report to considering sustainability Triple Bottom Line in project management activities. The purpose of this data article is to provide an understanding of the status quo and the trend for research on sustainability and project management in the world.

**Table:** Specifications table

Subject area	*Engineering*
More specific subject area	*Project Management*
Type of data	*Table, text file, graph, figure*
How data was acquired	*Scopus and Web of Sciences search analysis*
Data format	*Raw, filtered and analyzed data*
Experimental factors	*Search in Scopus and Web of Sciences conducted to obtain a global view of sustainability and project management research*
Experimental features	*Scientometric and bibliometric data*
Data source location	*Scopus and Web of Sciences data base*
Data accessibility	*Data is with this article*

**Table:** 

**Value of the data** • The set of scientometric data presented in the article allows researchers and other interested parties, to reach easily articles, authors and journals, as well as to present the views and tendencies of the main researchers working with sustainability and project management. • The dataset integrates recent and updated information with knowledge on sustainability and project management subject, adding more context to the data, providing support for future researches. • The dataset also provides information that will help to analyze the evolution of sustainability and project management research data in terms of co-word analysis, co-author analysis, journal analysis, institution analysis, and country analysis. • The dataset highlights the articles addressing sustainable project and project management methodology.

## 1 Data

The data is presented as a bibliometric dataset originated from 400 bibliographic records extracted, selected and refined from the Web of Science and Scopus core collection databases.

The data presents the evolution of the research field based on the concepts from the Brundtland Commission report to considering sustainability Triple Bottom Line in project management activities.

The scientometric data is presented trough combined information as tables, graphs and figures as networks and density maps, presenting a map of global research on sustainability and project management, and the key players on this subject area in 2006–2018: Fig. 1. Presents data on the evolution of the publication of research articles. Table 1. Searches executed on SCOPUS and WOS data bases; Fig. 2. Database refinement; Table 2. 17 most frequent words; Fig. 3. Most frequent words. Table 3. 17 most frequent co-occurring keywords; Fig. 4. Network of co-occurring keywords; Fig. 5. Item Density Visualization of co-occurring keywords; Table 4. The top 21 most productive authors; Table 5. The top 15 most productive co-authors; Fig. 6. Network Visualization of co-authorship; Table 6. The top 10 source journals; Table 7. The top more active institutions x country; Table 8. List of authors/articles x number of times the keyword codes (Green Project or Sustainable Project), were referenced; Table 9. List of authors/articles x number of times the keyword codes (Sustainable Project, Green Project, Project Management Methodology and Project Success), were referenced; Table 10. List of authors/articles x number of times the keyword codes (Lean Six Sigma, Project Management Methodology, Project Success and Sustainability), were referenced.

**Fig. 1:**
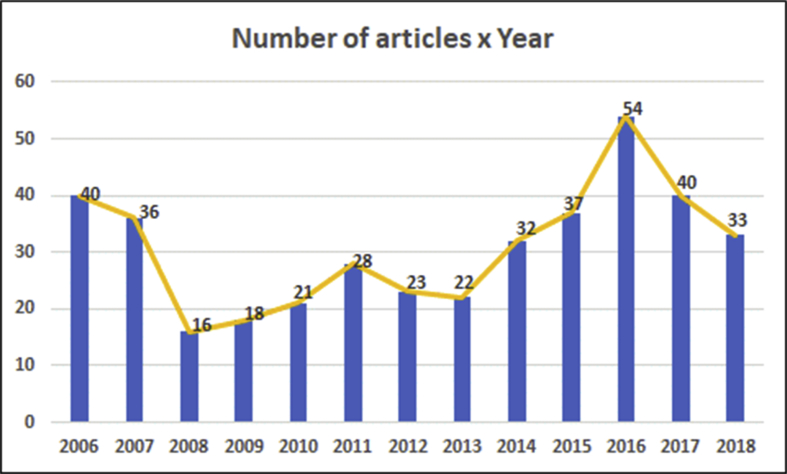
The number of articles on Sustainability and Project Management from WOS. and SCOPUS core collection after refinement in 2006–2018.

**Table 1:** Searches executed on SCOPUS and WOS data bases.

Search	Data Base	Keywords	Filters	Excluding Subjarea	Documents
1st	SCOPUS	"project management" AND ("methodology" OR "lean six sigma" OR "success" OR "green project")	→Document type: article →Language: English	MEDI" OR "AGRI" OR "CENG" OR "ARTS" OR "PSYC" OR "BIOC" OR "PHYS" OR "CHEM" OR "NURS" OR "HEAL" OR "PHAR" OR "IMMU" OR "VETE" OR "NEUR"	3929
2nd	WOS	"project management" AND ("methodology" OR "lean six sigma" OR "success" OR "green project")	→Document type: article →Language: English	INDUSTRIAL RELATIONS LABOR OR INSTRUMENTS INSTRUMENTATION OR ERGONOMICS OR MATHEMATICAL COMPUTATIONAL BIOLOGY OR NURSING OR METEOROLOGY ATMOSPHERIC SCIENCES OR ENGINEERING CHEMICAL OR PHARMACOLOGY PHARMACY OR HOSPITALITY LEISURE SPORT TOURISM OR PSYCHIATRY OR PSYCHOLOGY EXPERIMENTAL OR STATISTICS PROBABILITY OR SURGERY OR THERMODYNAMICS OR FOOD SCIENCE TECHNOLOGY OR AGRICULTURAL ECONOMICS POLICY OR BIOCHEMICAL RESEARCH METHODS OR CHEMISTRY MULTIDISCIPLINARY OR PSYCHOLOGY SOCIAL OR IMMUNOLOGY OR INTERNATIONAL RELATIONS OR AGRICULTURE MULTIDISCIPLINARY OR LINGUISTICS OR BIODIVERSITY CONSERVATION OR MINERALOGY OR ONCOLOGY OR FORESTRY OR PHYSICS APPLIED OR GEOSCIENCES MULTIDISCIPLINARY OR PSYCHOLOGY APPLIED OR RADIOLOGY NUCLEAR MEDICINE MEDICAL IMAGING OR SOCIAL ISSUES OR PSYCHOLOGY MULTIDISCIPLINARY OR SOCIAL SCIENCES BIOMEDICAL OR SOCIAL SCIENCES MATHEMATICAL METHODS OR SOCIAL WORK OR HEALTH CARE SCIENCES SERVICES OR SOCIOLOGY OR HEALTH POLICY SERVICES OR AGRONOMY OR VETERINARY SCIENCES OR ART OR VIROLOGY OR BIOTECHNOLOGY APPLIED MICROBIOLOGY OR ANESTHESIOLOGY OR MEDICAL INFORMATICS OR ARCHAEOLOGY OR CHEMISTRY ANALYTICAL OR BIOCHEMISTRY MOLECULAR BIOLOGY OR CHEMISTRY APPLIED OR HUMANITIES MULTIDISCIPLINARY OR CRIMINOLOGY PENOLOGY	1513
3rd	SCOPUS	"sustainability" AND ("green project" OR "triple bottom line" OR "carbon foot print" OR "global reporting initiative" OR "integrated reporting")	→Document type: article →Language: English	"AGRI" OR "ARTS" OR "BIOC" OR "CENG" OR "MEDI" OR "CHEM" OR "PSYC" OR "IMMU" OR "MULT" OR "HEAL" OR "NURS" OR "NEUR" OR "PHAR" OR "PHYS"	924
4th	WOS	"sustainability" AND ("green project" OR "triple bottom line" OR "carbon foot print" OR "global reporting initiative" OR "integrated reporting")	→Document type: article →Language: English	NURSING OR THERMODYNAMICS OR PSYCHOLOGY APPLIED OR AGRICULTURAL ENGINEERING OR GEOSCIENCES MULTIDISCIPLINARY OR AGRICULTURE DAIRY ANIMAL SCIENCE OR PUBLIC ENVIRONMENTAL OCCUPATIONAL HEALTH OR ART OR AGRICULTURAL ECONOMICS POLICY OR AGRICULTURE MULTIDISCIPLINARY OR CHEMISTRY MULTIDISCIPLINARY OR HOSPITALITY LEISURE SPORT TOURISM OR ERGONOMICS OR GEOGRAPHY PHYSICAL OR FORESTRY OR HISTORY OR GEOGRAPHY OR HISTORY PHILOSOPHY OF SCIENCE OR INDUSTRIAL RELATIONS LABOR OR INTERNATIONAL RELATIONS OR LAW OR BIODIVERSITY CONSERVATION OR POLITICAL SCIENCE OR SOCIAL SCIENCES INTERDISCIPLINARY OR STATISTICS PROBABILITY OR AGRONOMY OR PLANT SCIENCES OR BIOTECHNOLOGY APPLIED MICROBIOLOGY OR PSYCHOLOGY OR REMOTE SENSING OR SOCIOLOGY OR ENGINEERING CHEMICAL OR FOOD SCIENCE TECHNOLOGY OR SOIL SCIENCE OR SPORT SCIENCES	689
Total					7055

**Fig. 2:**
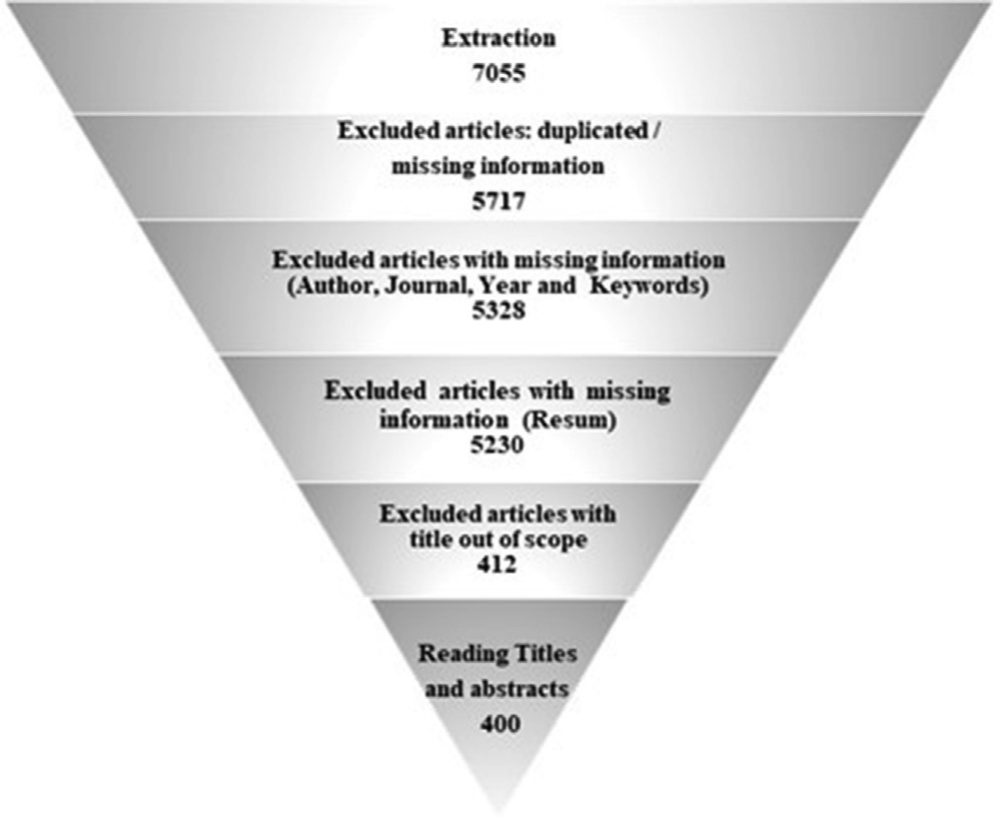
Database refinement (using EndNote®).

**Table 2:** 17 most frequent words (generated using NVivo®).

Words	Occurrence
sustainability	27560
management	18328
environmental	14711
project	12965
sustainable	11892
reporting	10003
business	9165
development	9055
research	8879
performance	8592
corporate	7288
economic	6421
companies	6040
analysis	5556
process	5534
information	5514
construction	5107

**Fig. 3:**
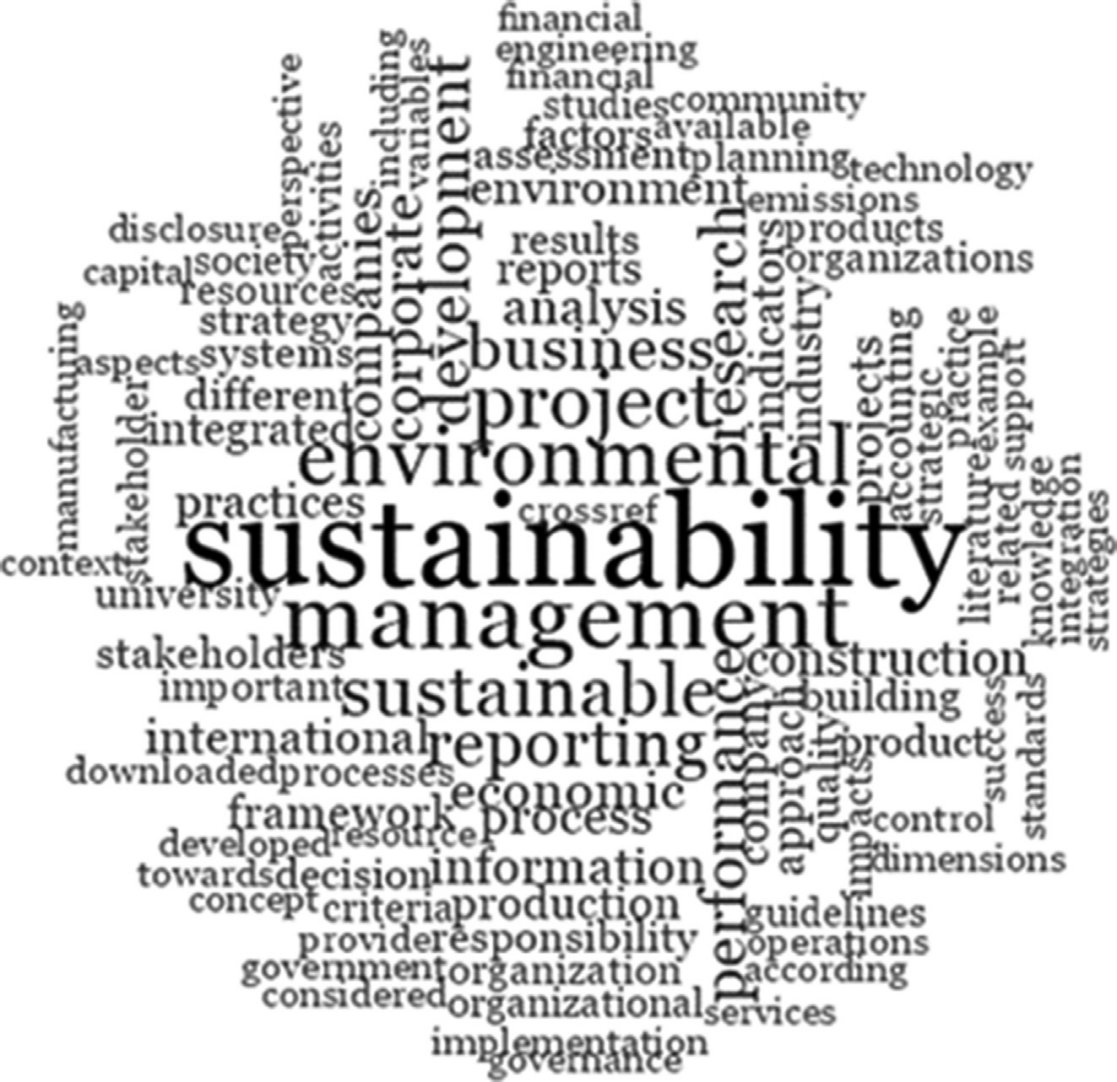
Most frequent words (“Word cloud view” - generated using NVivo®).

**Table 3:** 17 most frequent co-occurring keywords.

Keywords	Occurrence
sustainability	159
sustainable development	151
project management	100
triple bottom line	75
sustainability reporting	43
corporate social responsibility	38
construction industry	33
global reporting initiative	31
performance	30
decision making	27
supply chain management	25
environmental management	24
corporate sustainability	23
industry	22
environmental impact	21
management	20
integrated reporting	19

**Fig. 4:**
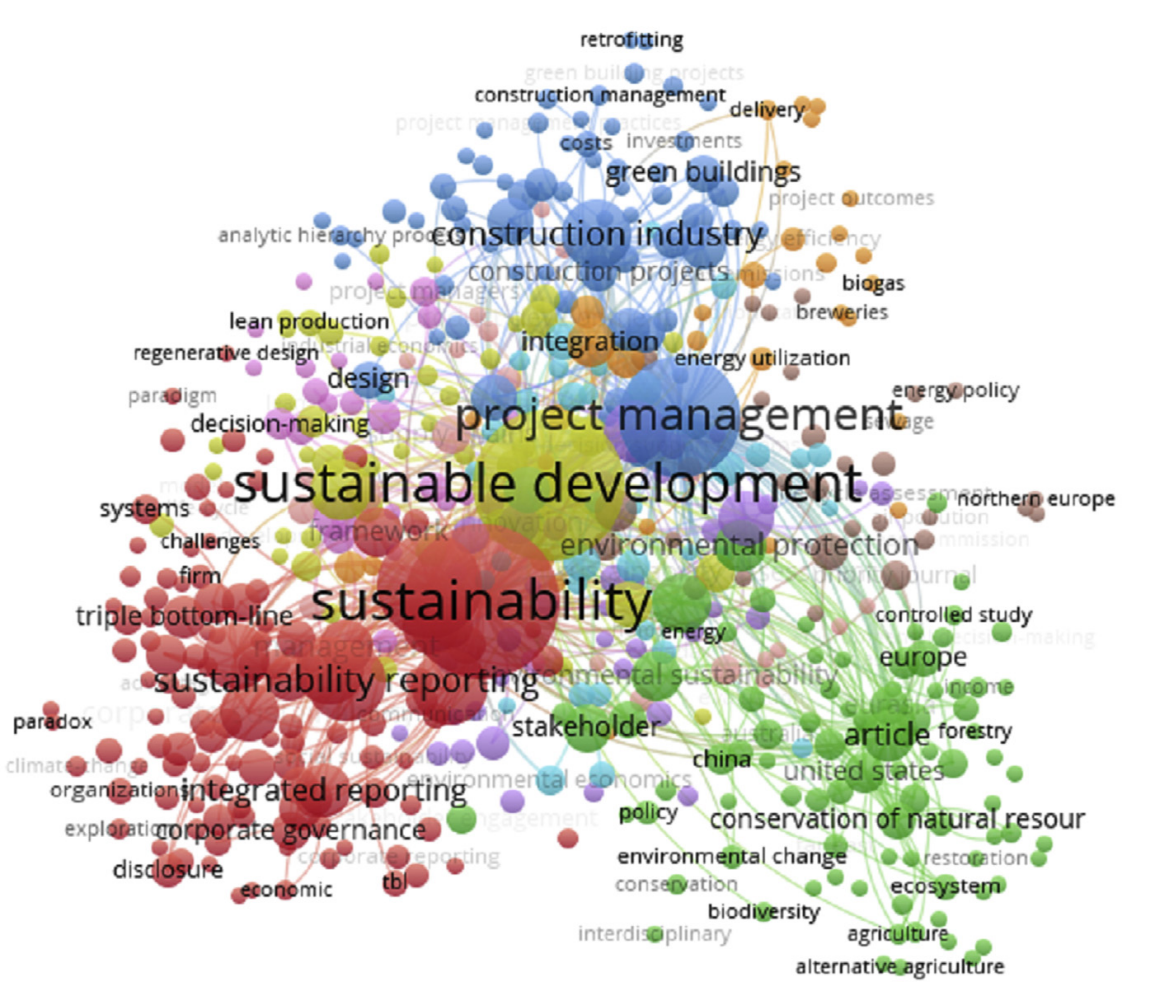
Network of co-occurring keywords.

**Fig. 5:**
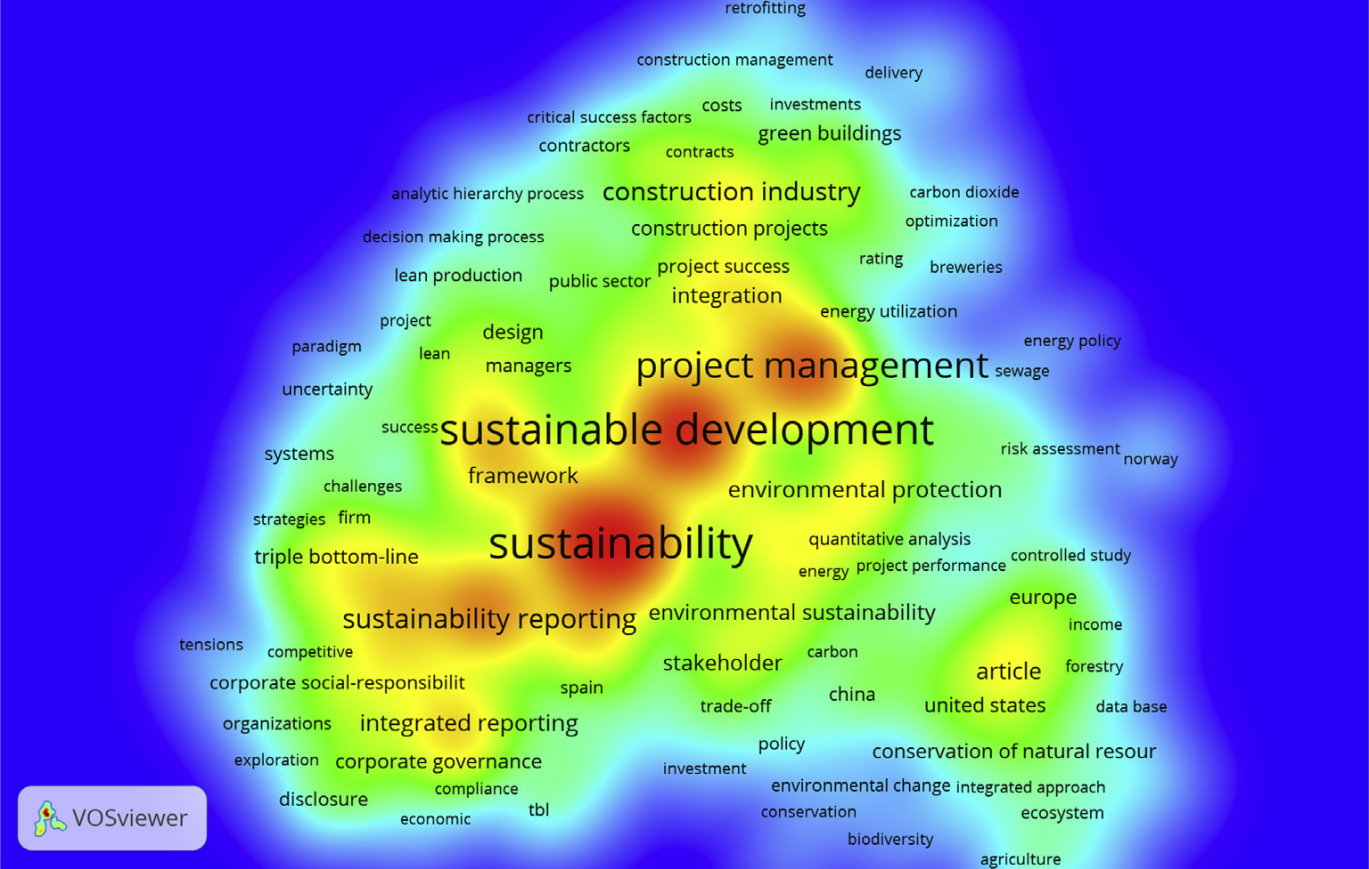
Item Density Visualization of co-occurring keywords.

**Table 4:** The top 21 most productive authors.

Author	Institution	Country	Count	Percentage
Silvius, G.	LOI University of Applied Sciences	Netherlands	3	0,8%
Isaksson, R.	Gotland University	Sweden	3	0,8%
Venkatraman, S.	University of Ballarat	Austrália	3	0,8%
Martens, M. L.	University of São Paulo	Brazil	3	0,8%
Svensson, G.	Kristiania University College	Norway	3	0,8%
Jamali, D.	American University of Beirut	Lebanon	2	0,5%
Lozano, R.	Cardiff University	United Kingdom	2	0,5%
Dittrick, P.	University of Texas at Austin	United States	2	0,5%
Gibson, K.	Marquette University, Wisconsin	United States	2	0,5%
Glass, J.	Loughborough University	United Kingdom	2	0,5%
Carter, C. R.	University of Nevada	United States	2	0,5%
Lam, P. T. I.	The Hong Kong Polytechnic University	Hong Kong	2	0,5%
Manetti, G.	University of Florence	Itália	2	0,5%
Smith, P. A. C.	The Leadership Alliance Inc.	Canadá	2	0,5%
Sridhar, K.	Macquarie University	Austrália	2	0,5%
Sarkis, J.	Clark University	United States	2	0,5%
Boiral, O.	Université Laval	Canadá	2	0,5%
Hwang, B. G.	National University of Singapore	Singapore	2	0,5%
Verrier, B.	University of Strasbourg	France	2	0,5%
Chofreh, A. G.	Buein Zahra Technical University	Iran	2	0,5%
Lucato, W. C.	University Nove de Julho	Brazil	2	0,5%

**Table 5:** The top 15 most productive co-authors.

Co-author	Institution	Country	Count	Percentage
Carvalho, M. M.	University of São Paulo	Brazil	4	1,0%
Nayak, R. R.	University of Ballarat	Austrália	3	0,8%
Searcy, C.	Ryerson University	Canadá	3	0,8%
Kumaraswamy, M.	University of Hong Kong	Hong Kong	2	0,5%
Meade, L. M.	Texas Christian University	United States	2	0,5%
Guthrie, J.	The University of Sydney	Australia	2	0,5%
Chan, E. H. W.	The Hong Kong Polytechnic University	Hong Kong	2	0,5%
Pagell, M.	York University	Canadá	2	0,5%
Seuring, S.	University of Kassel	Germany	2	0,5%
Rinaldi, L.	University of London	United Kingdom	2	0,5%
Svensson, G.	Kristiania University College	Norway	2	0,5%
Antunes, P.	University Nova de Lisboa	Portugal	2	0,5%
Rose, B.	University of Strasbourg	France	2	0,5%
Goni, F. A.	Islamic Azad University	Iran	2	0,5%
Melloni, G.	University of Verona	Italy	2	0,5%

**Fig. 6:**
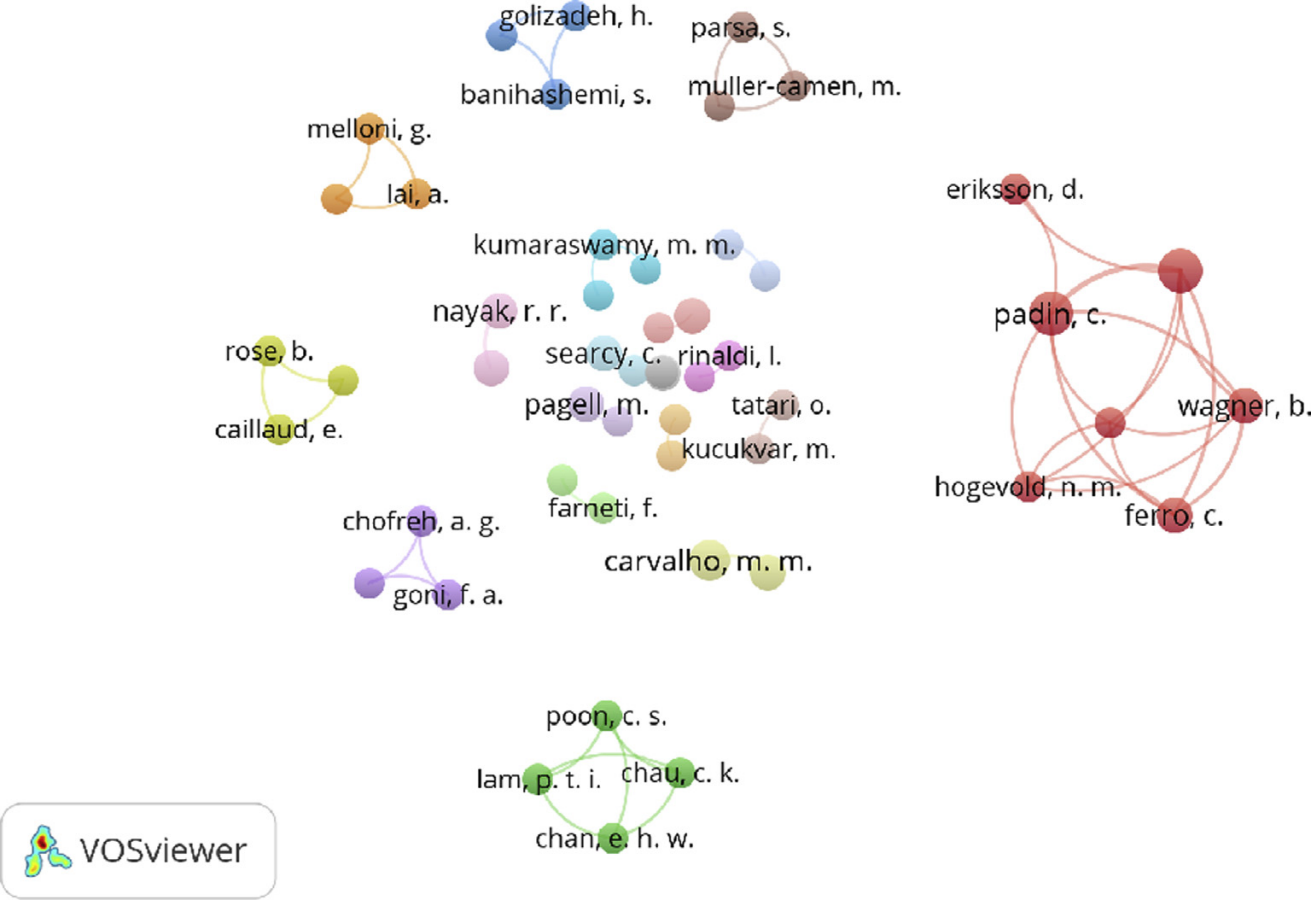
Network Visualization of co-authorship.

**Table 6:** The top 10 source journals for Sustainability and project management research in 2006–2018.

Journal	Country	Count	Percentage
Journal of Cleaner Production	Netherlands	38	9,5%
International Journal of Project Management	United Kingdom	13	3,3%
Business Strategy and the Environment	United States	12	3,0%
Environmental Management	United States	6	1,5%
Journal of Construction Engineering and Management	United States	6	1,5%
Sustainability	Switzerland	6	1,5%
Corporate Social Responsibility and Environmental Management	United States	5	1,3%
Engineering, Construction and Architectural Management	United Kingdom	5	1,3%
International Journal of Production Economics	Netherlands	5	1,3%
Journal of Management in Engineering	United States	5	1,3%

**Table 7:** The top more active institutions x country.

Institution	Country	Count
University of São Paulo	Brazil	4
LOI University of Applied Sciences	Netherlands	3
Gotland University	Sweden	3
University of Ballarat	Austrália	3
Kristiania University College	Norway	3
Ryerson University	Canadá	3
American University of Beirut	Lebanon	2
Cardiff University	United Kingdom	2
University of Texas at Austin	United States	2
Marquette University, Wisconsin	United States	2
Loughborough University	United Kingdom	2
University of Hong Kong	Hong Kong	2
Texas Christian University	United States	2
The University of Sydney	Austrália	2
The Hong Kong Polytechnic	Hong Kong	2
York University	Canadá	2
University of Kassel	Germany	2
University of London	United Kingdom	2

**Table 8:**
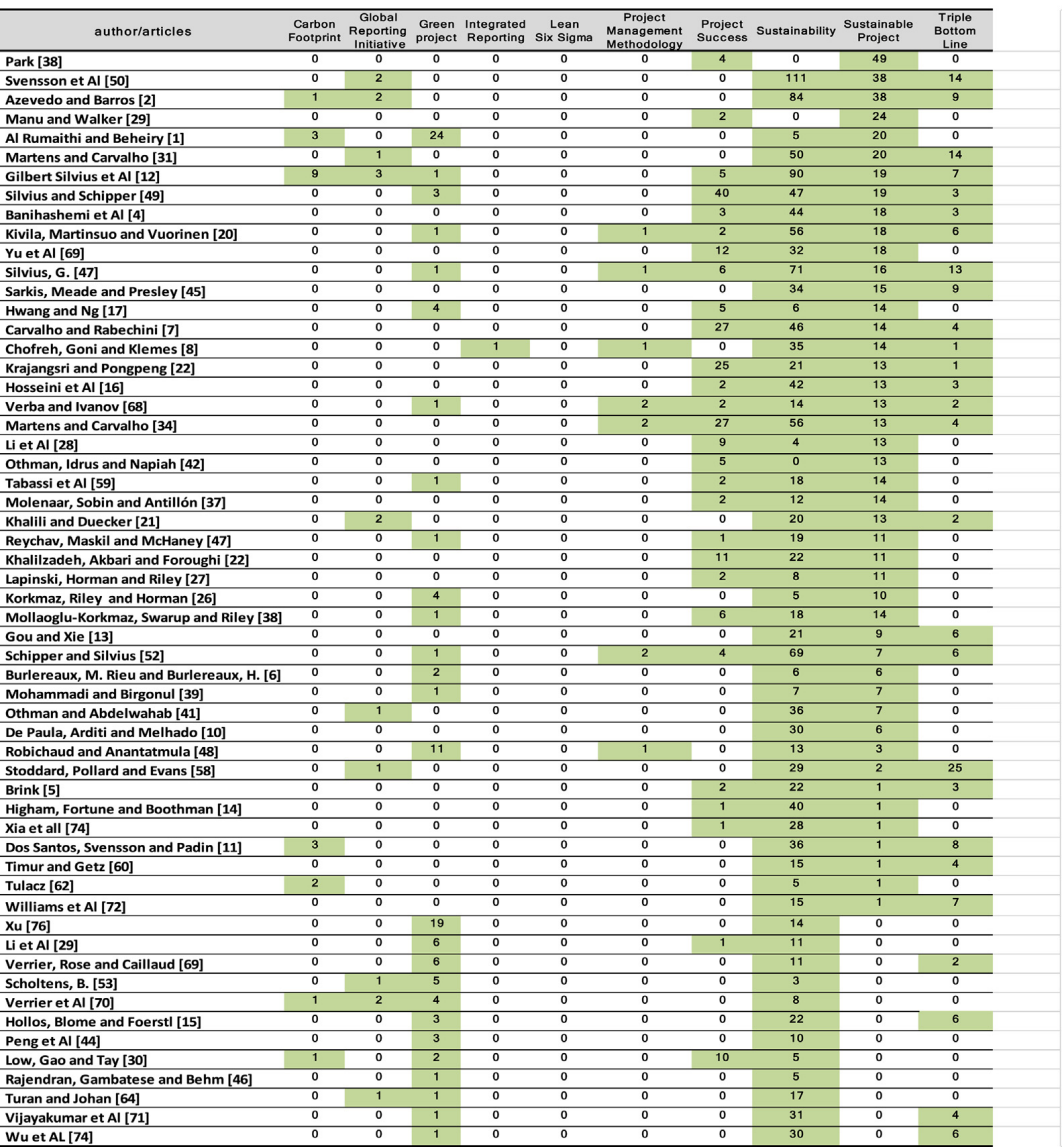
List of authors/articles x number of times the keyword codes (Green Project or Sustainable Project), were referenced.

**Table 9:**
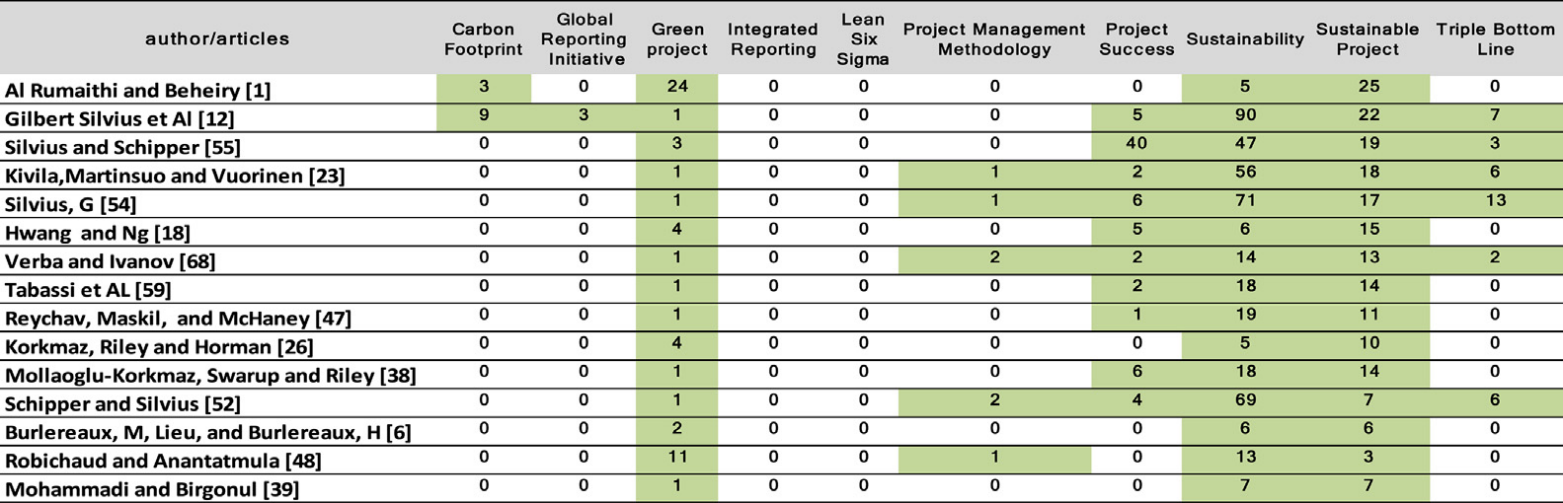
List of authors/articles x number of times the keyword codes (Sustainable Project, Green Project, Project Management Methodology and Project Success), were referenced.

**Table 10:**
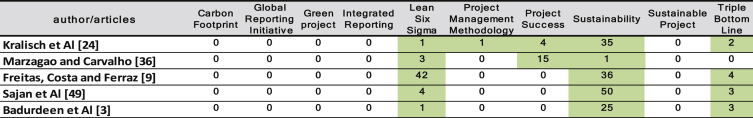
List of authors/articles x number of times the keyword codes (Lean Six Sigma, Project Management Methodology, Project Success and Sustainability), were referenced.

## 2 Experimental design, materials, and methods

### 2.1 Data collection

The majority of databases search realized, return a huge number of publications, in this work we've followed a methodology proposed by Treinta et al. [1]. After evaluating basic concepts related to sustainability and project management, the following retrieval codes were used on both database collections (Table 1.) to identify only document type articles written in English: 1- ("project management" and ("methodology" or "lean six sigma" or "success" or "green project") and 2- ("sustainability“ and ("green project“ or "triple bottom line“ or "carbon footprint“ or "global reporting initiative“ or "integrated reporting"). Were excluded from the data, articles from the following subject areas considered irrelevant to sustainability and project management: 1- SCOPUS ("medicine", "agriculture", "chemical engineering", "arts and humanities", “psychology", "biochemical", "physics", "chemistry", "nursing", "health", "pharmacology", "immunology", "veterinary", "neuroscience", and 2- WOS (industrial relations labor, instruments instrumentation, ergonomics, mathematical, computational biology, nursing, meteorology atmospheric sciences, engineering chemical, pharmacology pharmacy, hospitality leisure sport tourism, psychiatry, psychology experimental, statistics probability, surgery, thermodynamics, food science technology, agricultural economics policy, biochemical research methods, chemistry multidisciplinary, psychology social, immunology, international relations, agriculture multidisciplinary, linguistics, biodiversity conservation, mineralogy, oncology, forestry, physics applied, geosciences multidisciplinary, psychology applied, radiology nuclear medicine medical imaging, social issues, psychology multidisciplinary, social sciences biomedical, social sciences mathematical methods, social work, health care sciences services, sociology, health policy services, agronomy, veterinary sciences, art, virology, biotechnology applied microbiology, anesthesiology, medical informatics, archaeology, chemistry analytical, biochemistry molecular biology, chemistry applied, humanities multidisciplinary, criminology penology).

A total of 7055 articles were extracted and were exported and uploaded on EndNote® software, where we followed the following steps described on Fig. 2. in order to refine this database collection.

The Figures: 4- Network of co-occurring keywords; 5- Item Density Visualization of co-occurring keywords and 6- Network Visualization of co-authorship, and Table 3- 17 most frequent co-occurring keywords were generated using VOSviewer®.

At the end of this refinement process, a total of 400 bibliographic records were selected as the final database collection for considering in our analysis.

The EndNote software permits that a database collection be exported in different output styles. We've executed a complete database export in.xml form and imported it on Microsoft Excel. Microsoft Excel was used to support the data totalization and information formatting for articles x year, most productive authors, co-authors and top source journals. To support our bibliometric analysis, we've also selected NVivo - the most used qualitative and mixed-methods data analysis software tool and VOSviewer - a software tool for constructing and visualizing bibliometric networks. These networks may for instance include journals, researchers, or individual publications, and they can be constructed based on citation, bibliographic coupling, co-citation, or co-authorship relations.

With NVivo we executed the functions: a – “Word frequency”, to identify the most frequent words with minimum length of 7 letters, thus generating a “word cloud”; b – “Text search” for the same keywords used as retrieval codes for the databases, saving the data as codes for these keywords; c – “Matrix Coding”, to generate a matrix totalizing encountered items on the relation all authors x keyword codes; d – Selection and classification of “Matrix Coding” items that included Keywords code (Sustainable Project, Green Project, Project Management Methodology and Project Success), to identify authors x articles that referenced these codes.

### 2.2 Materials and methods

#### 2.2.1 Words and keywords frequency

This dataset took into account the words and keywords frequency in order to allow the comparison between terms used inside the full text with the authors keywords included on titles and abstracts.

For word frequency analysis, we've chosen NVivo software function “word frequency query”, selecting words with minimum length of 7 letters and restricting to display only the 1000 most frequent. The decision of choosing words with minimum length of 7 letters was taken, in order to avoid the inclusion of adverbs and pronouns on the data retrieved, prioritizing substantives and adjectives that are more representative of the data content. This function lists the most frequently occurring words or concepts, in our research we've applied over all articles PDF files. We can see this data on Table 2., it displays a list of the 17 most frequent words and the number of occurrences, and on Fig. 3. “Word cloud view”, it displays up to 100 words in varying font sizes, where frequently occurring words are in larger fonts.

For keywords frequency analysis, we've chosen VOSviewer software function “create a map based on bibliographic data”, + type of analysis; “co-occurrence” + unit of analysis: “keywords”, + counting method: “full counting”, + minimum number of occurrences of a keyword: “2”, and number of keywords to be selected: 500. The keywords are extracted from the titles and abstracts of each article. We can see this data on Table 3., 17 top keywords and number of occurrences and on Fig. 4. “Network of co-occurring keywords”. In co-occurrence analysis of keywords, the relatedness of items is determined based on the number of documents in which they occur together. The higher the number of co-occurrence of two terms, closed they will be located close to each other on the map.

In the network visualization, items are represented by their label and by default also by a circle. The size of the label and the circle of an item is determined by the weight of the item, this indicates the number of publications that have the corresponding term in their title or abstract. The higher the weight of an item, the larger the label and the circle of the item. For some items the label may not be displayed. This is done in order to avoid overlapping labels. The color of an item is determined by the cluster to which the item belongs. Van Eck NJ and Waltman L [2].

VOSviewer has grouped the terms into ten clusters, of which three are of significant size. The red cluster consists of sustainability terms. The yellow cluster covers terms related to sustainable development, the blue cluster consist of terms related to project management. The green cluster presents the keywords that appear less frequently.

On Fig. 5. We have “Item Density Visualization of co-occurring keywords”. In this visualization, colors indicate how nodes are distributed in the two-dimensional space underlying the visualization. The density visualization allows one to immediately identify dense areas in which many nodes are located close to each other. Van Eck NJ and Waltman L [4].

The larger the number of items in the neighborhood of a point and the higher the weights of the neighboring items, the closer the color of the point is to yellow. On the opposite, the smaller the number of items in the neighborhood of a point and the lower the weights of the neighboring items, the closer the color of the point is to blue. Van Eck NJ and Waltman L [4].

The keywords in red color area that appear more frequently: 1- sustainability, 2- project management and 3- sustainable development, each one pertains to the three higher clusters. These keywords are part of the core keywords in our research, we can also observe that sustainability is closer to sustainability reporting, integrated reporting and triple bottom line and project management is closer to construction projects and construction industry, but green buildings that pertains to the same cluster, has a lower weight.

#### 2.2.2 Author and Co-author

Table 4 presents the top 21 most productive authors. Silvius G. (LOI University of Applied Sciences), Isaksson R. (Gotland University), Venkatraman S. (University of Ballarat), Martens M. L. (University of São Paulo) and Svensson G. (Kristiania University College), occupied the top 5 positions.

Table 5 presents the top 15 most productive co-authors. Carvalho M. M. (University of São Paulo), Nayak R. R. (University of Ballarat) and Searcy C. (Ryerson University), occupied the top 3 positions.

A co-authorship network was generated using VOSviewer, for authors with a minimum of 2 articles, is presented in Fig. 6., where we could identify 72 authors, divided in 41 clusters or a research community. Each circle/node represents a researcher, large circles represent researchers that have many publications. Small circles represent researchers with only a few publications. In general, the closer two researchers are located to each other in the visualization, the more strongly they are related to each other based on bibliographic coupling. Van Eck NJ and Waltman L [3]. The colors indicate the strong collaboration stablished between the researchers as the circuit of Erikssom D., Ferro C., Hogevold N. M., Padin C., Svensson G., Valera J. C. S. and Wagner B., that is the largest community presented. The second largest community includes the researchers Chan E. H. W., Chau C. K., Lam P. T. I. and Pon C. S..

#### 2.2.3 Journals

Table 6 presents the top 10 source journals for sustainability and project management. Journal of Cleaner Production, published in Netherlands occupied the top position and published 38 articles (9,5%), followed by International Journal of Project Management (13 articles), published in United Kingdom and Business Strategy and the Environment (12 articles), published in United States. Out of the 10 journals, five journals are published in United States.

#### 2.2.4 Institution and country

Table 7 presents the contribution of Institutions and country from the 10 Top more productive authors and co-authors on sustainability and project management.

#### 2.2.5 Authors referencing the principal keywords code

Table 8, presents a total of 57 the records of authors/articles that referenced keywords code (Green Project or Sustainable Project).

Table 9, presents the authors/articles that referenced keywords code (Sustainable Project, Green Project, Project Management Methodology and Project Success), the only authors x articles that referenced all of these keywords together are: Kivila, J., Martinsuo M., Vuorinen, L. “Sustainable project management through project control in infrastructure projects” – 2017; Silvius G. “Sustainability as a new school of thought in project management” – 2017; Verba Y. S., Ivanov I. N. “Sustainable Development and Project Management: Objectives and Integration Results” – 2015 and Schipper R., Silvius G. “The sustainable project management canvas” – 2017.

Table 10, presents the authors/articles that referenced keywords code (Lean Six Sigma, Project Management Methodology, Project Success and Sustainability).
